# Evaluation of the Clinical, Technical, and Financial Aspects of Cost-Effectiveness Analysis of Artificial Intelligence in Medicine: Scoping Review and Framework of Analysis

**DOI:** 10.2196/33703

**Published:** 2022-08-12

**Authors:** Jesus Gomez Rossi, Ben Feldberg, Joachim Krois, Falk Schwendicke

**Affiliations:** 1 Department of Oral Diagnostics Digital Health and Health Services Research Charité–Universitätsmedizin Berlin Germany

**Keywords:** artificial intelligence, cost-effectiveness, systematic review, framework, health policy, research and development, cost, economics

## Abstract

**Background:**

Cost-effectiveness analysis of artificial intelligence (AI) in medicine demands consideration of clinical, technical, and economic aspects to generate impactful research of a novel and highly versatile technology.

**Objective:**

We aimed to systematically scope existing literature on the cost-effectiveness of AI and to extract and summarize clinical, technical, and economic dimensions required for a comprehensive assessment.

**Methods:**

A scoping literature review was conducted to map medical, technical, and economic aspects considered in studies on the cost-effectiveness of medical AI. Based on these, a framework for health policy analysis was developed.

**Results:**

Among 4820 eligible studies, 13 met the inclusion criteria for our review. Internal medicine and emergency medicine were the clinical disciplines most frequently analyzed. Most of the studies included were from the United States (5/13, 39%), assessed solutions requiring market access (9/13, 69%), and proposed optimization of direct resources as the most frequent value proposition (7/13, 53%). On the other hand, technical aspects were not uniformly disclosed in the studies we analyzed. A minority of articles explicitly stated the payment mechanism assumed (5/13, 38%), while it remained unspecified in the majority (8/13, 62%) of studies.

**Conclusions:**

Current studies on the cost-effectiveness of AI do not allow to determine if the investigated AI solutions are clinically, technically, and economically viable. Further research and improved reporting on these dimensions seem relevant to recommend and assess potential use cases for this technology.

## Introduction

The most widespread definition of artificial intelligence (AI) asserts that “It is the science and engineering of making intelligent machines, especially intelligent computer programs. It is related to the similar task of using computers to understand human intelligence, but AI does not have to confine itself to methods that are biologically observable” [1]. In the field of health care, AI is frequently referenced [2,3] as a tool [4] to improve diagnostics [5], facilitate screening [6], and optimize appointments for surgeries [7], among other use cases. Understanding these promising results requires considering AI research and development (R&D) as technically demanding and requiring consistent economic support for a long period of time. Some unique properties of AI, such as its high technical complexity and versatility of potential use cases, complicates studying AI solutions with standard cost-effectiveness analysis, which is frequent in the health care sector for pharmaceutical interventions. This in turn complicates judging its overall value by decision-makers [8-10].

Currently, aspects to define funding decisions for the R&D of AI, such as the success rate of these enterprises and the monetization strategies to incentivize these investments, remain understudied. We believe that this problem is to a degree due to a lack of frameworks that explicitly state these exclusive dimensions of AI analysis so that reporting of solutions can be made comparable, reproducible, and useful [11].

We hence developed the following theories:

1. Without clinical relevance (a clear value proposition for a health care stakeholder), AI solutions with a valid technical component (availability and annotation of data, software component, regulatory component, etc) and with a viable monetization strategy (an appropriate payment mechanism or model) could remain irrelevant to the health care system.
2. Without fulfilling technical requirements, clinically relevant AI tools with clear and promising financial potential could remain technically unfeasible.
3. Without sufficient monetization that justifies any development and recuperates any investment, clinically and technically feasible (and even desirable) AI solutions could be economically unviable.

Previous systematic literature reviews have analyzed the available body of evidence and have concluded that very few studies assessed the economic impact of AI with sufficient methodological rigor [12]. Importantly, no review to this date has looked at AI development through a comprehensive framework that relates the economic investment [13], the clinical impact, and the technical development of the technology, considering the cost of opportunity of investing in AI projects, which is standard in the pharmaceutical industry [14]. These factors have to be taken into account to improve our understanding of AI solutions and to assess the value added to patients by incorporating these solutions [15,16].

We conducted a systematic scoping review to assess existing literature from clinical, technical, and economic perspectives. We took a scoping approach to summarize the articles included in our review and constructed a framework that facilitates the comparison of AI R&D from these 3 perspectives, according to our above-discussed theories.

Scoping reviews are replicable and systematic, and are especially suited to assess an available body of evidence and to eventually inform research and policy priorities [17-19]. Frequently, they allow an exploratory research question to be framed within the available body of evidence to expose research gaps [19]. For the summary of our scoping review, we developed a health policy framework that merged and adjusted existing frameworks to this novel technology, according to existing best practice guides for health policy analysis [20]. These included exploring a new approach for synthesis and making our assumptions explicit, logical, interrelated, and open for empirical testing while focusing on synergizing with existing methods for analyzing AI technologies.

## Methods

### Synthesis and Reporting

Articles included in our scoping review were analyzed according to clinical, technical, and economic dimensions relevant to AI solutions, using our framework for analysis [21]. We consider an AI solution as any algorithm capable of classifying, recommending, analyzing, or suggesting the improvement of a clinical or organizational process without previous exposure to the data analyzed. We included AI solutions developed for a specific purpose, third-party AI solutions used as software as a service, and software solutions with an AI algorithm included in the service provided.

We then designed a framework for scoping and analyzing the literature included in our scoping review. This was achieved by combining different existing frameworks according to our proposed theory and extrapolating from them [20]. We then proceeded to adjust and quantify the categories applicable to this study. We then validated it by applying it to our research methods and proceeded to assess its saturation. The aim was to assess its usability, as well as determine which components were more frequently deployed in the field.

This review was conducted following the principles modified by Levac [18,22]. Reporting follows the PRISMA (Preferred Reporting Items for Systematic Reviews and Meta-Analyses) [Multimedia Appendix 1] 2020 statement [23]. This review could not be registered in the PROSPERO database since scoping reviews are not included from October 2019. The protocol entry is available from the authors on request.

### Eligibility Criteria

We included all forms of economic evaluations, reports on cost-effectiveness, and reports on the economic impact of AI solutions or AI algorithms used by any health care–related actor. Our population included patients, health care providers, insurance companies, the pharmaceutical industry, and suppliers of health care goods. Interventions would require the use of AI directly through a programing language that allows analyzing a certain database with a pre-existing open-source platform or data analysis library, as well as an integrated AI solution within a customized software. We preferred studies that compared AI against at least one comparator (ideally standard of care, ie, control), but we also assessed those without a control group, since new treatment paths or analyses may not have a clear comparator, such as in the case of fraud detection from an insurance perspective. Outcomes included in our review had a comparator of the utility, benefit, effectiveness, or cost assessed. No time limitation for the publication date was set. Our search was limited to English and German (the main languages spoken by our team).

### Information Sources, Search, and Study Selection

MEDLINE (via PubMed) and Embase (via Ovid) were searched for studies published until April 2021. Search strategies were adapted for each database. The following search strategy was used for PubMed: (((((((economic analysis[Title/Abstract]) OR (economic evaluation[Title/Abstract])) OR (cost-effectiveness[Title/Abstract])) OR (monetization[Title/Abstract])) AND (artificial intelligence[Title/Abstract])) OR (Convolutional neural network[Title/Abstract])) OR (machine learning[Title/Abstract])) OR (deep learning[Title/Abstract])

**Figure 1:**
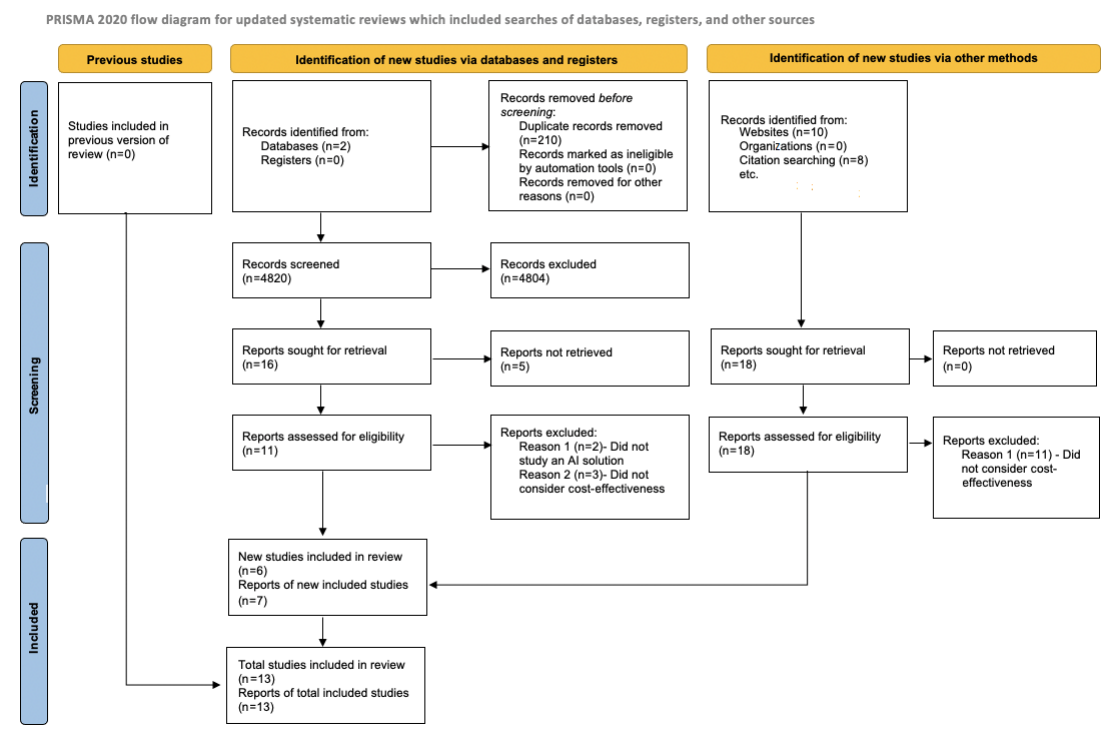
PRISMA (Preferred Reporting Items for Systematic Reviews and Meta-Analyses) 2020 [23] flowchart. AI: artificial intelligence.

Two reviewers (JGR and BF) independently screened the identified studies for eligibility. Potentially eligible studies were assessed (JGR and BF), and inclusion was decided in consensus with a third reviewer (FS). We created a list of cited sources and then proceeded to manually retrieve the sources and evaluate in full the articles for inclusion. When this second source lead to a third source and the third source met the inclusion criteria, this source was also included and classified as “citation research” in our analysis. In order to expand our scope, a hand search was performed online to look for scientific references on regulatory AI databases mentioned in the studies included, and these were included as studies identified from “websites.” Details can be seen in Figure 1. The decision of not including specific medical disciplines in the search strategy was deliberate and aimed at achieving a broad inclusion of articles tailored to medical databases.

### Inclusion/Exclusion Criteria

The inclusion criteria for AI studies were as follows: (1) a solution developed using AI or any technique encompassed on it (machine learning, deep learning, etc); (2) application to any medical field/medical facility directly providing services to patients, and (3) any claim over a cost-effectiveness analysis of this technology, regardless of the methodology utilized.

The exclusion criterion was grey literature to accommodate for the lack of a risk of bias assessment in scoping reviews.

### Data Collection Process and Items

Data extraction was performed by 2 reviewers independently (JG and BF) in a pilot-tested spreadsheet. Eligible studies were collected in a single spreadsheet for screening, which was performed by each reviewer independently. Studies meeting the inclusion criteria were marked by each reviewer and accepted after comparing results with those of the other reviewer. Disagreements were solved by consensus-based discussion, and if infructuous, they were solved by consulting a third reviewer (FS).

The following data were collected: year and country where the study was conducted, what outcomes were measured and how, payer’s perspective assumed, comparator considered (if applicable), what benefits were measured, and what analysis was used to compare differences in the effect with the baseline case. When applicable, the following data were collected: who annotated the data for training the algorithm, how was the data set composed, image type or information type used to train the algorithm, use case assumed, AI algorithm used, and diagnostic accuracy considered (sensitivity/specificity).

### Data Synthesis and Framework Construction

Our policy framework was developed according to Walt et al [20] and Hetrick et al [24] to synthesize our included articles. According to our theory, the framework for synthesis considered the following 3 dimensions: clinical aspects, technical aspects, and monetization/economic aspects. To analyze these dimensions, 2 authors (JGR and BF) proceeded to generate 3 independent lists (1 per dimension) to include in the analysis and rank the articles by completeness, correctness, and logic consistency for the analysis of AI. Selected frameworks were then adjusted and presented to a third reviewer (FS) who assisted in maintaining the development of the tool within the scope of our research theory by having the final vote over discrepancies and presenting alternatives where evidence was not easily available.

We desisted from using tools to assess the risk of bias or assess methodological quality since scoping reviews do not aim to produce a critically appraised and synthesized result/answer to a particular question. They rather aim to provide an overview or map of the evidence. Due to this, assessing methodological limitations or risk of bias of the evidence included within a scoping review is generally not performed [17]. The PRISMA checklist for systematic scoping reviews was utilized.

The generated framework can be found in Multimedia Appendix 2.

#### Clinical Consideration of AI R&D

First, AI solutions were classified in the medical discipline they are supposed to be designed for (part 1a), according to the typology developed by the Association of American Medical Colleges [25] and modified to include dentistry. We did so to achieve a broader coverage of all medical disciplines contained in this framework, since dentistry may not be within the jurisdiction of medical colleges but instead dental colleges, and despite that belongs to the health care field. The categories were allergy and immunology, anesthesiology, dermatology, diagnostic radiology, emergency medicine, public health, internal medicine, medical genetics, neurology, nuclear medicine, obstetrics and gynecology, ophthalmology, pathology, pediatrics, physical medicine and rehabilitation, psychiatry, radiation oncology, surgery, urology, and dentistry.

Second, we considered the perspectives of users (1b), defined as those who use or benefit from using AI solutions. In health care, differences exist between those who use a service (reflected in this classification) and those who pay for it (analyzed in the economic considerations of this framework; 3b). The typology for this classification was extracted from the work of Sneha et al [26] who categorized value propositions in eHealth. The categories were as follows: patients, health care professionals, insurance companies, pharmaceutical companies, and vendors or suppliers.

Third, the value proposition (1c), that is, the benefit derived from using AI solutions, was analyzed. Our classification was derived from the frameworks for software and mobile health published by Gorski et al [27] and Walther et al [28], who defined value propositions for software and modified them to include AI. The categories were as follows: improved experience for users or professionals, improved data collection/curation, outsourcing of screening to another provider, improved financing, optimized direct resource utilization (medical resources utilized for the process optimized: capital or labor), optimized indirect resources (waiting times, detecting possible cancellation of appointment, etc), branding, fraud detection/quality control, risk assessment, improved recommendation of a provider/product, community building and transparency, accounting benefits, energy savings, replacement of old infrastructure (outsourcing of processes), improved data security, improved mobility, improved availability, improved ease of use, improved helpdesk quality (follow-up of cases and chatbots), facilitation of innovation, improved actualization of a service or product, and strategic flexibility (lower sunk costs).

As an “AI value proposition,” we added optimization, which we defined as improved output with the same resources or reduced costs producing the same output. We acknowledge that this classification may require revision over time as AI unravels new value for users.

Finally, AI solutions were grouped (1d) according to the “EU software as medical device regulation (MDR)” from 2017 [29], using a binary categorization to assess the need for premarket approval. Because the exact determination of risks is normally independently evaluated by regulatory bodies, this classification is purposefully general to differentiate AI solutions that could be part of medical devices and affect pathways of care from those use cases that would not require market preapproval by regulatory bodies. The categories were “No” or “Class I/II/III” (needing premarket approval).

#### Technical Aspects of AI R&D

Developing AI solutions in health care could be particularly demanding as regulatory bodies require, in some cases, extensive testing of these products before granting them market approval. As a result, AI investors could expect high costs to enter the market and a lower success rate. It is expected that AI investors take the perspective of pharmaceutical companies and expect that the benefits from a successful digital product compensate for the high failure rate of other projects [30-34]. This is a common practice in the pharmaceutical field [35]. As a result, the framework assesses the direct R&D costs per AI solution generated that successfully enters the market (2a) and the R&D costs of the jointly developed products that do not enter the market (2b).

The direct costs per AI solution generated (2a) were divided into the 2 categories of labor and capital. We considered the following as “fixed” direct costs of AI development (paid once): data generation/acquisition, data labeling, data science, software engineering services, overheads (marketing, management, and hardware), and regulatory costs.

The costs of R&D for the pharmaceutical industry from an investor perspective (2b) comprise the costs of investing in the development of an AI solution, adjusted by the risk of failure. In this industry, previous studies have estimated the costs per new product brought to the market considering both direct and indirect (personnel and overhead) R&D costs per therapeutic product each year, adjusted by inflation to US$ using the US consumer price index [36]. Other studies have retrospectively assessed the cost of the opportunity of investing in pharmaceutical products by assessing all projects managed by a pharmaceutical company, including those that failed, and dividing total R&D costs by the costs for projects that succeeded, to conclude the “cost of money” for these enterprises, as the real cost of capital rate has been historically 10.5% per year [14]. This allows the estimation of the required risk-free rate of return for an investor considering other investment opportunities, paid as a yearly premium [37,38]. It is likely that R&D costs of AI included in our framework would lead to significant underestimation since, to this date, there is insufficient information on the return of investment on AI in health care.

The “variable” costs or costs per good sold grow with output (2c). They were estimated based on a real-world AI solution in dentistry [39] (Multimedia Appendix 3), which considered exclusively cloud infrastructure and customer support. Although this assumption is likely to underestimate other running costs, such as improvement of the algorithm, marketing, and surveillance, among others, it seeks to make explicit that some commercial use cases of AI require a dedicated postmarket launch team that could be later added to the section “Others” of our framework.

The categories we assessed consisted exclusively of “cloud infrastructure,” “customer support and quality management,” “third-party products,” and “other costs.”

#### Monetization of AI

This dimension explicitly analyses AI solutions’ payment mechanisms (3a) and payment model (3b). It should be noted that the potential beneficiaries and users of AI solutions are more diverse than the narrow patient perspective taken to analyze clinical outcomes in standard pharmacological products. Payment mechanisms are derived from an analysis of the value of data by Deighton et al [40]. To make possible cross-country comparisons, as well as comparisons across different use cases, we focused exclusively on payment methods irrespective of the legislation of the country we were assessing. Because there are many major differences in access to the market by different products, the results should be interpreted with caution. A certain business model dependent on a monetization scenario may likely be highly impactful, cost-effective, and profitable in one setting, but completely irrelevant, not very cost-effective, or completely illegal when extrapolated to another. Because of that, this category exclusively focuses on naming options for monetization found in the literature while remaining open to incorporating future monetization scenarios. We acknowledge that for AI developers, decisions on how and where to access a market will be conditional on a complete evaluation of a legal landscape rapidly changing and not considered in this review.

The categories in the payment mechanisms analyzed included the following: license or white labeling, one-time purchase, freemium and premium, SaaS (assuming a flat fee for each service provided), publicity, pay-for-performance, profit sharing, shared saving, bundled payment, and exclusivity contract.

An appropriate payment model is a requisite for a sound business model in digital health [41]. As discussed previously, this category helps to assess explicitly who is supposed to pay for the solution and in which contractual modality, and not who benefits from the AI solution. This category differentiates between AI solution companies focused on offering services to other companies (known as “business to business” or “B2B”) and companies focused on offering the same AI services but to individual consumers (“business to consumer” or “B2C”).

### Risk of Bias

All classifications were carefully evaluated by the reviewer team (JGR, FS, and BF), and disagreements were solved by discussion. Further quantitative synthesis or evaluation of meta-biases was not feasible due to high data heterogeneity. The risk of bias or the assessment of methodological quality was not included in this review since scoping reviews do not aim to produce a critically appraised and synthesized result/answer to a particular question, as discussed [17].

## Results

### Included Studies and Data Description

**Figure 2:**
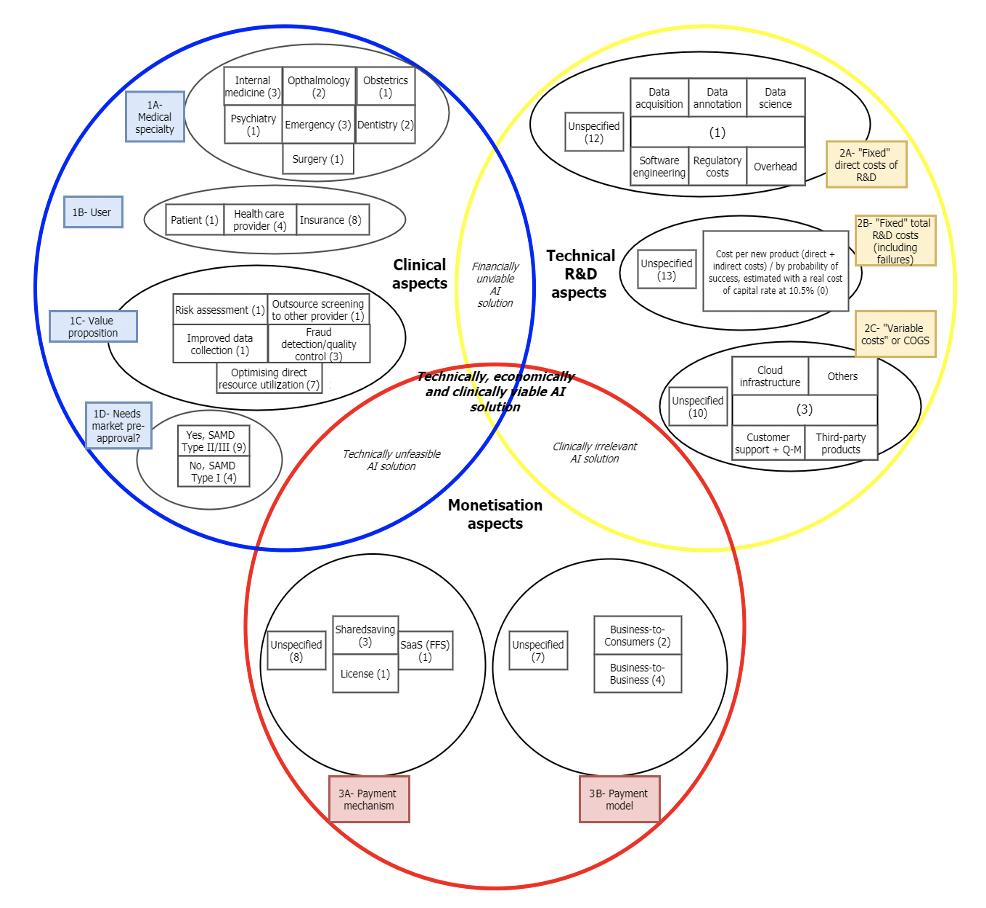
Mapping of identified studies along with the developed framework. AI: artificial intelligence; COGS: costs per good sold; R&D: research and development; SAMD: software as a medical device.

Mapping of the identified studies is presented in Figure 2. Studies were grouped according to the clinical, technical, and economic aspects of the AI. Each article was categorized in our framework according to the pre-established categories extracted from the literature. When the information necessary for classification was not available, the corresponding AI solution was classified as “unspecified.” AI solutions could only be included at 1 level.

We identified 4820 articles as initially eligible for our review through database screening. After screening, 16 studies were retrieved and assessed in full, and 6 were included in this review (Figure 1). Additionally, 7 other studies were included after being identified via a web search and citation search. The studies excluded did not meet the criterion of considering the dimensions of cost-effectiveness in the AI solutions they analyzed. Three systematic literature reviews met the inclusion criteria posed by our review. Two of them were conducted by a governmental body that aimed to find cost-effective therapies for diagnostic screening.

Multimedia Appendix 4 summarizes the articles meeting the inclusion criteria [39,42-53]. The studies included showed broad variability in the data used by the AI solution to generate inference and in the types of algorithms used, and frequently compared their results to the standard of care. Among the 13 studies included, 5 (39%) took place in the United States, 2 (16%) took place in Germany, and 2 (16%) took place in Canada, and Singapore, Turkey, Zambia, and the United Kingdom had 1 (7%) study each.

The majority of the studies included (9 of 13) assessed AI solutions that may require some form of premarket authorization. Internal medicine and emergency remained the most frequently studied specialties. AI solutions aimed at patients and health care providers were studied in 5 cases. The optimization of direct resource use remained the most frequent value proposition (7/13, 54%).

The technical aspects analyzed remained unaddressed to a large degree. Except for 3 (23%) studies, most of the articles analyzing the cost-effectiveness of AI solutions disregarded variable costs. Only 1 study estimated fixed costs of R&D, disregarding any reporting on opportunity costs from an investor’s perspective. No study considered the costs of data acquisition or failed enterprises.

The economic aspects analyzed remained underreported to a significant degree. Furthermore, even among those studies in which a payment model was assumed (6/13, 46%), the payment mechanism could not be identified in the use case analyzed. A minority of articles that analyzed the cost-effectiveness of AI explicitly stated the payment mechanism assumed (5/13, 38%), with a majority (8/13, 62%) insufficiently reporting the mechanism.

## Discussion

### Principal Findings

AI is a novel yet highly promising and versatile technology with a demonstrated capacity to undertake different tasks in the medical field with high accuracy, and unlike standard pharmaceutical products, it can help different actors of the health care system in a variety of different use cases. However, compared with other fields, standard cost-effectiveness evaluations may require adaptations, which is why this study developed a common framework for evaluation and to facilitate communication between developers, patients, doctors, and decision-makers.

The use of our framework fosters a comprehensive assessment of different dimensions of AI and makes explicit assumptions involving AI R&D, frequently overseen in previous studies. We believe that having these in mind can help to optimize research solutions where they can have the most impact by considering appropriate budgeting. Importantly, this framework could as well give both decision-makers and developers common ground to negotiate payment methods by explicitly stating the costs of development.

The analysis of our results using our framework indicates that the majority of the economic evaluations included in our study reported the clinical or organisational benefits of AI without an appropriate disclosure and justification of technical and financial aspects that substantiate these claims. It is likely that a relevant share of information and aspects is hence not fully reflected, possibly leading to biased conclusions by these studies. This seems relevant because it possibly calls for an improvement in reporting AI R&D, especially in the area of costs surrounding technical and monetization aspects to facilitate recognizing the niches where AI development will have the highest benefit for society.

Our results also invite further consideration of the setting of analysis, as regulation and market access may vary greatly and determine the economic viability of AI solutions. More transparent disclosure of clinical, technical, and economic aspects could not only generate common ground to differentiate promising projects from those excessively technically complex or clinically irrelevant, but also simplify the cooperation between AI developers, investors, clinicians, patients, and regulators.

### Strengths and Limitations

First, in this review, we did not comprehensively assess the qualitative aspects of the included studies, such as their risk of bias. This limitation seemed acceptable in light of our initially planned scope, which was focused on developing a framework of analysis to gauge the comprehensiveness and completeness of existing studies. Second, although our framework could require extension with further categories of analysis and future adjustments, we believe it has succeeded in making explicit the current research gaps in the existing body of literature. Third, we acknowledge the lack of other comprehensive frameworks of analysis and limited evidence supporting our analyses, which is why this article should be considered as the start of the scientific analysis of the cost-effectiveness of AI in health care. We acknowledge that our conclusions are preliminary in a field that continues to evolve rapidly, and our results should be interpreted with caution, as future methods of analysis will have to be developed jointly with new AI solutions.

Future studies could validate or disprove the completeness of this framework and possibly work to continue and reform some of its components as AI technology continues to expand its functionality over time. Additionally, future scoping reviews could help to obtain an overview of the development of this technology over time and help to identify suitable comparisons between subfields involving AI, which could greatly facilitate generating systematic literature reviews focused on clinical effectiveness, such as meta-analyses and formal cost-effectiveness comparisons.

### Conclusion

The literature reviewed in our study was sparse and did not seem comprehensive enough to draw a conclusive analysis on AI's potential to facilitate cost-effective healthcare. While some studies have showcased the positive impact of AI adoption, future research should improve reporting of the technical aspects of AI development. This seems important to achieve better comparisons of similar use cases of this novel and highly versatile technology. We believe that the adoption of the framework discussed in this study can facilitate more robust scientific analysis and better-informed conclusions on the potential of this technology.

## Appendix

Multimedia Appendix 1 PRISMA-ScR Checklist.

Multimedia Appendix 2 Clinical, technical, and economic dimensions included in our framework for analysis.

Multimedia Appendix 3 Input parameters for dentistry.

Multimedia Appendix 4 Articles included in the review.

## Abbreviations

AI: artificial intelligence
B2B: business to business
B2C: business to consumers
COGS: cost per good sold
MDR: medical device regulation
R&D: research and development
SAMD: Software as a medical device
