# Supplementary material for: Evaluation of the Clinical, Technical, and Financial Aspects of Cost-Effectiveness Analysis of Artificial Intelligence in Medicine: Scoping Review and Framework of Analysis
Source: JMIR Med Inform. 2022 Aug 12;10(8):e33703. doi: 10.2196/33703 (PMC9419048; doi:10.2196/33703)
Supplement: Multimedia Appendix 2 [file medinform_v10i8e33703_app2.docx]

**Multimedia Appendix 1.** Clinical, technical, and economic dimensions included in our framework for analysis.

| 1. Clinical aspects of **the AI solution** | | | | 1. **R&D and operational costs** | | | 1. **Strategy of monetisation** | |
| --- | --- | --- | --- | --- | --- | --- | --- | --- |
| **Medical specialty [25)** | **Users perspective** [26] | **Value proposition** [27,28] | **Pre-approval SAMD** [29] | **Direct costs of developing AI** [39] | **Details on total R&D costs** [36] | **COGS** [39] | **Payment mechanism** [40] | **Payment model** [41] |
| **Immunology** | Patients (P) | Improved experience | No | Data generation/ acquisition | Direct and indirect costs disclosed for all AI projects in the same portfolio at a  the real cost of capital rate of 10.5% | Cloud infrastructure | License or white labelling | Business-to-Business (B2B) |
| **Anesthesiology** | Healthcare Professionals (HCP) | Improve data collection / Curation | Yes (Class I/ II/III) | Data labelling |  | Customer support | One time purchase | Business-to-Consumers (B2C) |
| **Dermatology** | Insurances (I) | Expand indication for screening |  | Software engineering |  | Others | Freemium and premium |  |
| **Diagnostic radiology** | Pharmaceuticals (Pharm) | Improved financing |  | Overhead (Marketing etc.) |  |  | SaaS (Software as service) |  |
| **Emergency** | Suppliers (Sup) | Optimise direct resource utilisation |  | Regulatory approval |  |  | Pay for performance |  |
| **Public health** |  | Optimise indirect resource utilisation |  | Hardware for training |  |  | Profit-sharing |  |
| **Internal medicine** |  | Improve branding |  | Hardware for data acquisition |  |  | Shared saving |  |
| **Medical genetics** |  | Fraud detection / quality control |  |  |  |  | Bundled payment |  |
| **Neurology** |  | Risk Assessment |  |  |  |  | Exclusivity contract |  |
| **Nuclear medicine** |  | Recommendation of provider / service |  |  |  |  |  |  |
| **Gynecology/Obstetrics** |  | Improve transparency |  |  |  |  |  |  |
| **Opthalmology** |  | Improve actualisation of processes |  |  |  |  |  |  |
| **Pathology** |  | Improve accounting |  |  |  |  |  |  |
| **Paediatrics** |  | Replacement of infrastructure |  |  |  |  |  |  |
| **Physical medicine** |  | Improve data security |  |  |  |  |  |  |
| **Psychiatry** |  | Improve mobility |  |  |  |  |  |  |
| **Radiation oncology** |  | Helpdesk quality (Follow-up/ chatbots) |  |  |  |  |  |  |
| **Surgery** |  | Facilitate innovation |  |  |  |  |  |  |
| **Urology** |  |  |  |  |  |  |  |  |
