# Supplementary material for: Evaluation of the Clinical, Technical, and Financial Aspects of Cost-Effectiveness Analysis of Artificial Intelligence in Medicine: Scoping Review and Framework of Analysis
Source: JMIR Med Inform. 2022 Aug 12;10(8):e33703. doi: 10.2196/33703 (PMC9419048; doi:10.2196/33703)
Supplement: Multimedia Appendix 3 [file medinform_v10i8e33703_app3.docx]

**Multimedia Appendix 2.** Input parameters for dentistry.

| **Prevalence, accuracy, lesion development and progression** | | | | | |  | | |  | | | |  |
| --- | --- | --- | --- | --- | --- | --- | --- | --- | --- | --- | --- | --- | --- |
| **Estimate** | | **Source (reference)** | | | **Initial lesions (up to  inner third of enamel) (E2)** | **Initial lesions (up outer third of dentin) (D1)** | | | | **Advanced lesions  (middle third of dentin) (D2)** | | |  |
| **Prevalence** | | | |  | | | |  | | | |  |  |
| Low risk | Schwendicke et al. (23) | | | | 0.14 | 0.025 | | | | 0.005 | | | |
| High risk | Schwendicke et al. (23) | | | | 2.14 × 0.14 | 1.66 × 0.025 | | | | 1.66 × 0.005 | | | |
| **Sensitivity and specificity** | | | |  | | | |  | | | |  |  |
| Sensitivity  Visual-tactile | Schwendicke et al. (23) | | | | 0.00 | 0.00 | | | | 0.311 (0.270-0.353) | | | |
| Specificity  Visual-tactile | Schwendicke et al. (23) | | | | 1.00 | 1.00 | | | | 0.922 (0.892-0.945) | | | |
| Sensitivity  Radiography w/o AI (control)* | Schwendicke et al. (38) | | | | 0.24 (0.21-0.26) | 0.36 (0.24-0.49) | | | | 0.64 (0.59-0.70) | | | |
| Specificity  Radiography w/o AI (control)* | Schwendicke et al. (38) | | | | 0.97 (0.95-0.98) | 0.94 (0.89-0.97) | | | | 0.98 (0.97-0.98) | | | |
| Sensitivity  Radiography w AI (test) | Garcia Cantu et al. (39) | | | | 0.68 | 0.68 | | | | 0.58 | | | |
| Specificity  Radiography w AI (test) | Garcia Cantu et al. (39) | | | | 0.86 | 0.86 | | | | 0.96 | | | |
| Probability of being tested | KZBV- Statutory Guideline (46) | | | | 2 per year | 2 per year | | | | 2 per year | | | |
|  |  | | | |  |  | | | |  | | | |
| Probability of lesion development | Schwendicke et al. (23) | | | | p=1.26 × 0.57252 × 2.7^-0.1472 × 2α^ distribution: 1.24-1.29 | p=1.26 × 0.0426 × 2.7^-0.0521 × 2α^  distribution: 1.24-1.29 | | | | p=1.26 × 0.57 × 0.0426 × 2.7^-0.0521 × 2α^  distribution: 1.24-1.29 | | | |
|  |  | | | |  |  | | | |  | | | |
| Probability of lesion progression | | |  | | | |  | | | |  |  |  |
| Progression to |  | | | | D1 lesion | D2 lesion | | | | D3 lesion | | | |
| If untreated | schwendicke et al. (23) | | | | p=2.63 (high risk) / 2.13 (low risk) × 3.0984×(2α)^-1.343^  (distribution: p × 0.87 – p × 1.13) | p=2.63 (high risk) / 2.13 (low risk) × 161.52×(2α)^-2.078^  (distribution: p × 0.87 – p × 1.13) | | | | p=1.32 × 161.52 ×  (2α)^-2.078^  (distribution: p × 0.87 – p × 1.13) | | | |
| If infiltrated | Schwendicke et (23) | | | | p=0.4289×(2α)^-1.391^  (distribution: p × 0.23 – p × 5.15) | p=68.869×(2α)^-2.078^  (distribution: p × 0.23 – p × 4.17) | | | | - | | | |
| **Transition probabilities** | | | | |  |  | | | |  | | | |
| **Health state** | **Source (reference)** | | | | **Transition probability per cycle** | **Transition to** | | | | **Allocation probability** | | | |
| Composite^11^ | (Pallesen et al.) | | | | 0.016 | Composite  Crown  Repair  Rootcanal treatment  Extraction | | | | 0.45  0.10  0.10  0.25  0.10 | | | |
| Direct capping^2^ | Schwendicke et al. (47) | | | | 0.111 | Rootcanal treatment  Extraction | | | | 0.95  0.05 | | | |
| Crown on vital tooth^3^ | Burke and Lucarotti (48) | | | | 0.036 | Rootcanal treatment  Recementation  Repair  Re- crown  Extraction | | | | 0.25  0.15  0.10  0.40  0.10 | | | |
| Root canal treatment | Lumley et al. (49) | | | | 0.021 | Non-surgical re-treatment Surgical re-treatment Extraction | | | | 0.20  0.30  0.50 | | | |
| Crown on non-vital tooth^3^ | Burke and Lucarotti (48) | | | | 0.029 | Recementation  Repair  Re- crown^3^  Extraction | | | | 0.20  0.10  0.60  0.10 | | | |
| Non-surgical root-canal treatment | Ng et al. (50) | | | | 0.085(Ng et al. 2008) | Surgical re-treatment  Extraction | | | | 0.25  0.75 | | | |
| Surgical root-canal treatment | Torabinejad et al. (51) | | | | 0.061 | Extraction | | | | 1.00 | | | |
| Implant and implant-supported crown | Torabinejad et al. (52) | | | | 0.010 | Recementation/refixing Re-crown  Re-implant | | | | 0.60  0.20  0.20 | | | |

^1^ Data from 15-19-year-olds. The risk of pulpal exposure during re-composite assumed to be 10%. Crowning assumed if re-restored before.

^2^ 95% of exposed pulps were treated using direct capping, 5% were assumed to receive immediate root canal treatment.

^3^ For non-vital crowned teeth, the risk of endodontic complications was calculated separately (Ferrari et al. 2012).

Analyses were performed for populations with low and high caries prevalence and risks, respectively. Sensitivities and specificities of caries detection with and without AI assistance were derived from our primary study and a meta-analysis as described. The probabilities of lesion development and progression if untreated or infiltrated were calculated according to the patient’s age (α) using hazard functions. If possible, we calculated mean values and 95% confidence intervals or ranges to estimate distributions (in parentheses) for random sampling during microsimulation.
