# Supplementary material for: Evaluation of the Clinical, Technical, and Financial Aspects of Cost-Effectiveness Analysis of Artificial Intelligence in Medicine: Scoping Review and Framework of Analysis
Source: JMIR Med Inform. 2022 Aug 12;10(8):e33703. doi: 10.2196/33703 (PMC9419048; doi:10.2196/33703)
Supplement: Multimedia Appendix 4 [file medinform_v10i8e33703_app4.docx]

**Multimedia Appendix 3.** Articles included in the review.

| Setting/Year | | Clinical aspects of the AI solution analyzed | | | | R&D and operational costs of the AI solution analyzed | | | Strategy of monetization | | Technical aspects analyzed in the paper | | | |
| --- | --- | --- | --- | --- | --- | --- | --- | --- | --- | --- | --- | --- | --- | --- |
| Country (Study) | Year | Specialty | User | Value proposition | Market approval needed? | Direct costs of creating AI | R&D costs including failed enterprises | COGS | Payment mechanism | Payment model | Algorithm | Research question | Data used | Comparator |
| UK [42] | 2020 | Internal medicine | HCP | Improving data collection/curation | Yes (Class I/ II/III) | N/S | N/S | N/S | N/S | N/S | Deep Learning | Are increased monitoring and real-time analysis of information cost-saving? | Heart rate | Standard of care |
| USA [43] | 2018 | Surgery | HCP | Optimizing direct resource utilization | Yes (Class I/ II/III) | N/S | N/S | N/S | N/S | N/S | SegNet/ ENet | Is producing the same product with cheaper open-source algorithms cost saving? | CT/MRI | Licensed software |
| Canada [44] | 2019 | Emergency medicine (SLR) | Ins | Optimizing direct resource utilization | Yes (Class I/ II/III) | N/S | N/S | N/S | N/S | N/S | No articles matched the search criteria | What is the cost-effectiveness of portable stroke diagnostic devices for patients with stroke symptoms? | N/S | Standard of care |
| Canada [45] | 2020 | Internal medicine (SLR) | Ins | Optimizing direct resource utilization | Yes (Class I/ II/III) | N/S | N/S | N/S | N/S | N/S | No articles matched the search criteria | What is the cost-effectiveness of artificial intelligence for nodule classification in screening, incidental identification, or known or suspected malignancies for lung cancer? | CT | Radiologists |
| USA [46] | 2019 | Emergency medicine | Ins | Risk assessment | No | N/S | N/S | N/S | N/S | N/S | RUSBoost | Can machine learning outperform logistic regression in predicting resource utilization by patients with total joint replacement? | 33 Risk factors | Logistic regression |
| Turkey [47] | 2018 | Internal medicine | Ins | Fraud detection/ quality control | No | N/S | N/S | N/S | License | B2B | Unspecified (included in software AlinIQ) | Do auditing and overriding of physician decisions reduce costs? | Tests requested by physicians | Physicians unaudited |
| Germany [48] | 2018 | Psychiatry | Ins | Optimize direct resource utilization | Yes (Class I/ II/III) | N/S | N/S | N/S | Unclarified, presumably shared saving | B2B | Support vector, regression tree, ridge regression | Can machine learning generate cost-effective recommendations on therapies for patients under treatment for depression? | Demographic data, current treatment, MINI Neuropsychiatric interview, quick inventory of depressive symptomatology, Patient health questionnaire, 5-levelEQ-5D, Costs, Treatment preferences | Standard of care |
| Zambia [49] | 2019 | Obstetrics | Ins | Fraud detection/ quality control | No | N/S | N/S | N/S | Unclarified, presumably shared saving | B2B | Random forest, Naïve Bayes, Logistic Regression, Support Vector Machines | Can methods of machine learning be used cost-effectively to optimize auditing to detect over-reporting in performance-based financing schemes in Zambia? | Insurance claims from hospitals | Auditing hospitals by random sampling |
| USA [50] | 2018 | General dentistry | Ins | Fraud detection/ quality control | No | N/S | N/S | N/S | N/S | N/S | Unsupervised Markov renewal process | Are preventive services resulting cost-effective in reducing dental care utilization? | Insurance claims public insurance, surveys | Children that did not receive preventive dental care |
| USA [51] | 2018 | Emergency | HCP | Optimizing Direct resource utilization | Yes (Class I/ II/III) | N/S | N/S | N/S | N/S | N/S | Deep unified networks (DUNs), logistic regression, gradient boosting, maxout networks | What is the diagnostic accuracy of some machine learning methods to detect the risk of 30-days readmission of patients with heart failure discharged from the hospital after an episode in the US? | Feature vectors included structured demographic, utilization, and clinical data, as well as selected extracts of unstructured data from clinician-authored notes | Patients discharged alive with standard auditing methods |
| Singapore [52] | 2020 | Ophthalmology | Ins | Optimizing direct resource utilization | Yes (Class I/ II/III) | N/S | N/S | IT costs all-inclusive: S$15 | Shared saving * | B2B | Multiscale feature bank detector and CNN | What testing strategy is cheapest, provided that all generate the same results? | Retinal fundus images | Semi-teleophthalmology screening strategy and technician-based diagnosis |
| USA [53] | 2020 | Ophthalmology | P | Expand indication for screening | Yes (Class I/ II/III) | N/S | N/S | Set as 0 us$ | N/S | B2C | Multiscale feature bank detector and CNN | What is the cost per True-positive of artificial intelligence in diabetic retinopathy as point of care diagnostic? | Retinal fundus images | Screening exclusively by ophthalmologists |
| Germany [39] | 2020 | General dentistry | HCP | Optimizing direct resource utilization | Yes (Class I/ II/III) | Data acquisition: not included, Data labelling: Included, Data science: Included, Software engineering: Included, Overhead: included; Total: 239 918 Euros | N/S | Cloud infrastructure: Included, Support: included, Others: Not included// Total: 2.65 Euros/Image | SaaS (FFS) | B2C | CNN | What is the cost-effectiveness of artificial intelligence for proximal caries detection as decision-support system? | Dental x-rays (bitewings) | Dentists |

B2B: Business-to-Business, B2C: Business-to-Consumer, CT: Computed tomography, CNN: Convolutional neural networks, N/S: Not-Specified, P: Patient, HCP: Healthcare Provider, Ins: Insurance, MRI: Magnetic resonance, SaaS (FFS): Software-as-Service (Fee-for-service).
